# The Role of the Transcription Factor EGR1 in Cancer

**DOI:** 10.3389/fonc.2021.642547

**Published:** 2021-03-24

**Authors:** Bin Wang, Hanfei Guo, Hongquan Yu, Yong Chen, Haiyang Xu, Gang Zhao

**Affiliations:** ^1^ Department of Neurosurgery, The First Hospital of Jilin University, Changchun, China; ^2^ Cancer Center, The First Hospital of Jilin University, Changchun, China

**Keywords:** EGR1, cancer, proliferation, apoptosis, angiogenesis, invasion, migration, metastasis

## Abstract

Early growth response factor 1 (EGR1) is a transcription factor that is mainly involved in the processes of tissue injury, immune responses, and fibrosis. Recent studies have shown that EGR1 is closely related to the initiation and progression of cancer and may participate in tumor cell proliferation, invasion, and metastasis and in tumor angiogenesis. Nonetheless, the specific mechanism whereby EGR1 modulates these processes remains to be elucidated. This review article summarizes possible mechanisms of action of EGR1 in tumorigenesis and tumor progression and may serve as a reference for clinical efficacy predictions and for the discovery of new therapeutic targets.

## Introduction

**Figure 1:**
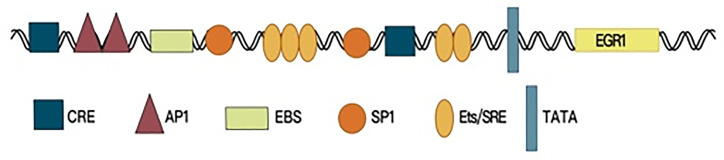
The promoter region structure of EGR1.

Early growth response factor 1 (EGR1) is a member of the EGR family and is also known as EGR-1, NEFI-A, Zif268, Krox-24, and TIS85. The *EGR1* gene is located in human chromosomal region 5q23-31, and the protein is an important transcription factor (1), which contains an activation regulatory region, repressive regulatory region, and three Cys2-His2 subclass zinc finger structures, which specifically recognize and bind target genes and regulate their transcription. The *EGR1* promoter region contains serum response elements. A variety of growth factors can start *EGR1* gene expression by interacting with this sequence, which contains the characteristic motif CC(A/T)_6_GG, namely, the CArG box (2, 3). Furthermore, the *EGR1* promoter contains an EGR1-binding sequence (EBS), which forms a negative feedback loop to control *EGR1* expression (4, 5) (**Figure 1**).

EGR1 is widely expressed in many cell types and participates in important physiological processes, such as cell proliferation, differentiation, invasion, and apoptosis. When cells are stimulated by growth factors, tumor necrosis factor, inflammatory factors, ionizing radiation, reactive oxygen species, or other factors (6–9), EGR1 can be activated through the MAPK signaling pathway. *EGR1* transcription depends on the RAS–RAF–MEK1/2–ERK1/2 signal transduction pathway, and activated EGR1 can either cause or inhibit the expression of its target genes, thus playing a part in transcriptional regulation (10).

As a transcription factor, EGR1 performs a regulatory function in cell growth, but the roles of EGR1 are different in different tumors. For example, EGR1 expression is higher in a prostate tumor than in surrounding prostate tissue (11), and the expression of EGR1 in the prostate tumor positively correlates with malignancy (12). Similar clinical associations have been found in gastric cancer (13). The expression of EGR1 is significantly higher in a primary gastric tumor and metastases than in normal gastric tissues, and EGR1 expression correlates with tumor size, depth of invasion, tumor stage, and prognosis (14). Therefore, EGR1 plays an oncogenic part in prostate and gastric cancers. Nonetheless, EGR1 upregulates tumor suppressor gene *p21^Waf1/Cip1^* and leads to tumor cell apoptosis in gliomas and melanocytomas (15, 16), and consequently, EGR1 serves as a tumor suppressor in these cancers.

EGR1 also plays a dual role in different signaling pathways. EGR1 suppresses transformation and counteracts apoptosis *via* coordinated activation of TGF-β1, FN, p21^Waf1/Cip1^, and FAK, thereby leading to enhanced cell attachment and reduced caspase activity (17). On the other hand, in cells with decreased adhesion, EGR1 enhances PTEN-mediated downregulation of AKT expression, thus increasing apoptosis (18). EGR1 regulates the attachment and survival of normal cells but induces apoptosis in abnormal cells with decreased adhesion.

Therefore, EGR1 performs important functions in tumor cell proliferation, angiogenesis, invasion, and immune responses (19–21). Research on the mechanism of action of EGR1 in cancer is expected to point to a new cancer treatment strategy and/or a new marker for theranostics. The purposes of this review are to summarize the roles of EGR1 in tumor cell proliferation, apoptosis, and metastasis and in the tumor microenvironment as well as to discuss the possible signaling pathways in which EGR1 is involved to provide a new perspective on cancer treatment.

## The Oncogenic Effects of EGR1

**Figure 2:**
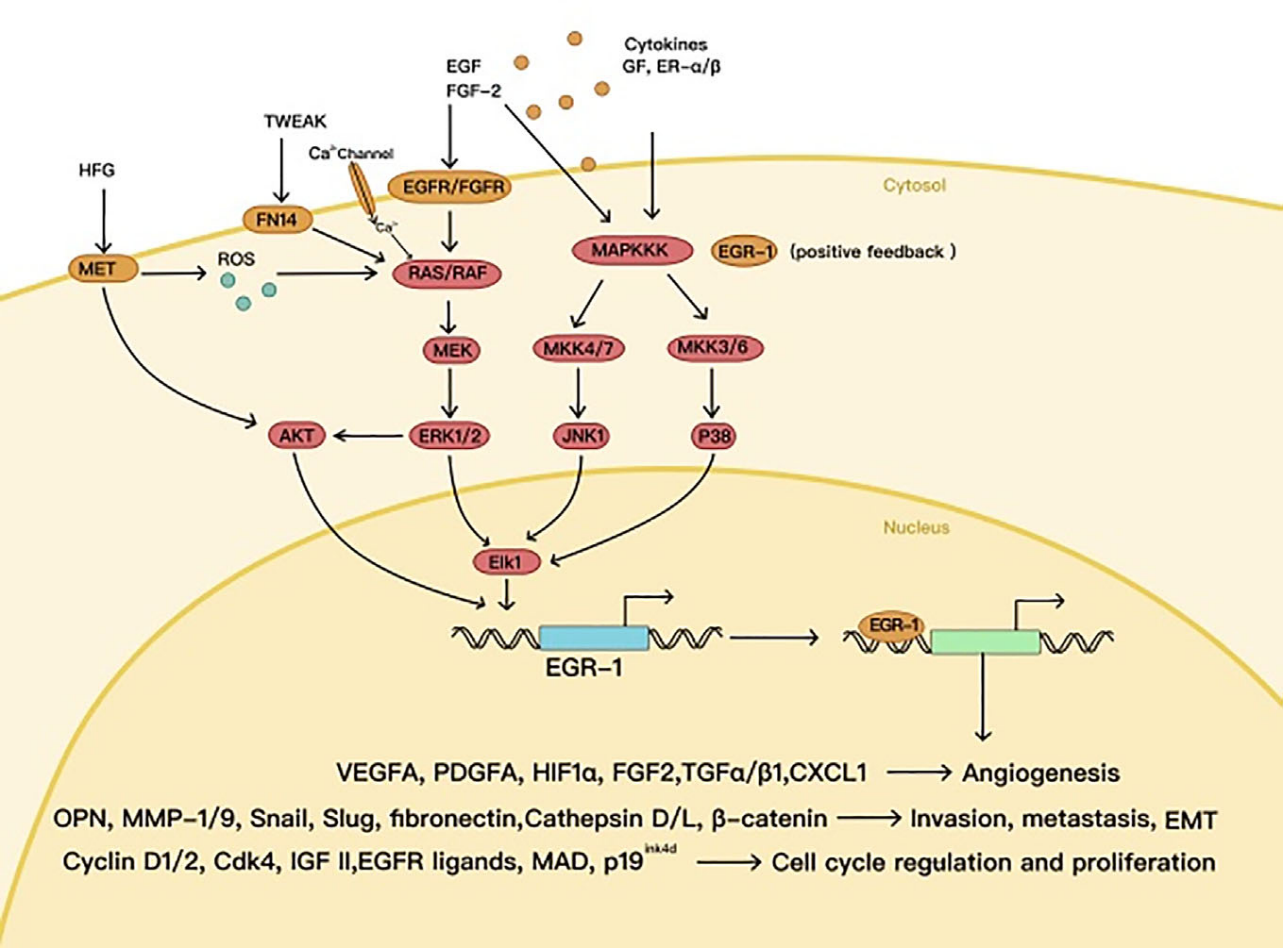
The oncogenic effects of EGR1.

High expression of EGR1 has been observed in various tumors, in which EGR1 can play an oncogenic role, e.g., glioma, lung cancer, gastrointestinal tumors, and melanoma (22–25). As a downstream protein of the MAPK signaling pathway (26), EGR1 can promote transcriptional activation of cyclin D1 in many tumor types and maintain the mitosis of tumor cells (27). Meanwhile, EGR1 contributes to tumor metastasis as well, by starting SLUG and SNAIL expression (28, 29). Under hypoxic conditions, EGR1 increases VEGFA expression, mediated by HIF1α, and directly activates VEGFA transcription (30), thus promoting the formation of blood and lymphatic vessels in the tumor (31) (**Figure 2**).

## The Roles of EGR1 in Cell Cycle Regulation and Tumor Cell Proliferation

Various authors have shown that high EGR1 expression may increase the proliferation of specific types of tumor cells by affecting the cell cycle. For example, the high expression of EGR1 in prostate cancer is a potential precancerous event. The level of the EGR1 protein in prostate cancer tissue positively correlates with the Gleason score, which predicts the tumor proliferation ability, and negatively correlates with the differentiation degree of prostate cancer cells (32). These data indicate that EGR1 participates in prostate cancer progression (33). There has been a similar finding in gastric cancer: patients whose tumor is histologically classified as malignant exhibit higher tumor EGR1 levels and a stronger ability of tumor cells to proliferate (24).

The MAPK–ERK pathway is a classic proliferation signaling cascade that is triggered by growth factors. In C6 cells, estrogen receptor β (ERβ) can launch the RAF–MEK1–ERK–ELK1 signaling pathway and upregulate EGR1 (10). EGR1 expression decreases significantly after treatment with PD98059, a MAPK–ERK pathway inhibitor, suggesting that *EGR1* is a downstream gene of the MAPK–ERK pathway (26). Through inhibition of MAPK phosphorylation, the nuclear concentration of EGR1 can be reduced while the proliferation of breast cancer cells can be significantly suppressed (34). Cyclin D1 is necessary for the progression of the G1 cell cycle phase and can shorten this phase, whereas the RAS–RAF–MEK–ERK pathway plays an important role in the initiation of cyclin D1 gene expression (35, 36). EGR1 activated by the MAPK cascade can directly bind to the cyclin D1 promoter, strengthen the expression of cyclin D1, and then may result in cell proliferation by advancing cells from the G1 phase to S phase (27). EGR1 is also able to induce tumor cell proliferation by upregulating other cell cycle–related proteins such as cyclin D2 and CDK4 (28). Overexpressed EGR1 may directly trigger the p38 MAPK signaling pathway too (24). In summary, the mechanism behind the interaction between EGR1 and the MAPK–ERK signaling pathway is a positive feedback process. EGR1 overexpression not only promotes tumor growth but also launches the p38 MAPK signaling pathway. The EGR1-activated MAPK–ERK signaling pathway further enhances EGR1 expression, and the upregulated EGR1 accelerates cell proliferation by controlling the expression of cyclin-dependent kinases (CDKs) (24, 27, 28, 33).

## The Participation of EGR1 in Tumor Invasion and Metastasis

Epithelial–mesenchymal transition (EMT) means a morphological change of epithelial cells to mesenchymal cells, which is an important mechanism of tumor cell invasion and metastasis. EGR1 contributes to tumor invasion and metastasis mainly by starting the expression of E-cadherin transcriptional inhibitors (SNAIL and SLUG). In other words, SNAIL and SLUG can inhibit the expression of E-cadherin, and EGR1 plays an important role in tumor EMT by regulating SNAIL and SLUG. In hormone-independent prostate cancer, CXCL5 (also known as ENA78) enhances EGR1 transcription *via* the RAF–MEK–ERK pathway, thus increasing SNAIL expression, tumor cell metastasis, and EMT (28). In hepatocellular carcinoma cells, EGR1 induced by hepatocyte growth factor (HGF) can directly bind to the promoter region of *SNAIL*, increase its expression, and lead to tumor cell metastasis (37). In ovarian cancer cells, epidermal growth factor (EGF) has been shown to induce *EGR1* and upregulate SLUG, which can decrease the expression of E-cadherin and then enhance tumor metastasis (29). In hepatocellular carcinoma, EGR1 induces *SLUG* through the ERK–AKT–EGR1–SLUG signaling cascade and stimulates EMT of cancer cells (38).

Additionally, interstitial-space–related genes, such as matrix metalloproteinase 1 (*MMP1*), *MMP9*, cathepsin, and zinc finger-binding homeobox 1 (*ZEB1*), play an important part in tumor metastasis. EGR1 can directly bind to the promoter region of *MMP1* and trigger its expression. SNAIL may enhance the expression of interstitial-space–related genes, such as *MMP9* and *ZEB1*. A variety of MAPK pathways, such as ERK1 and -2, JNK, and p38 kinase cascades, participate in the expression of MMP1 caused by tumor necrosis factor alpha (TNF-α) by upregulating EGR1 and promote tumor invasion and metastasis (39). EGR1 can synergistically act with SNAIL on the promoter regions of *MMP9* and *ZEB1*, thereby enhancing their transcription and initiating tumor cell invasion and metastasis (40). In oral squamous cell carcinoma, overexpression of hTERT activates tumor invasiveness by raising the expression of cathepsin D *via* EGR1 (41). EGR1 is also known to stimulate EMT of non–small cell lung cancer (NSCLC) cells through the mut-p53–EGR1–cathepsin L signaling cascade (42). EGR1 can directly bind to the promoter sequences of a variety of interstitial-space–related genes to start their expression and therefore performs an important function in tumor invasion and metastasis.

In gastric cancer, EGR1 has been reported to enhance tumor cell proliferation and invasion by increasing β-catenin expression (43). In breast cancer, S100A4 drives the nuclear localization of EGR1 by promoting the binding of EGR1 to importin 7, and EGR1 next enhances tumor invasion and metastasis by downregulating β-catenin through the PTEN–AKT–GSK3β signal transduction (44). Consequently, EGR1 may control β-catenin expression and enhance tumor invasion and metastasis, but the specific mechanism is yet to be revealed.

## The Roles of EGR1 in Tumor Angiogenesis

Tumor angiogenesis is an important mechanism of tumor growth and metastasis, and many achievements in the treatment of cancers have been made through inhibition of tumor angiogenesis. Due to the rapid growth of a tumor, its central area is often in a state of hypoxia. The latter is a potent angiogenesis-stimulating factor. Hypoxia-inducible factors (HIFs) are upregulated by hypoxic conditions and increase the expression of vascular endothelial growth factor (VEGF) family proteins. Furthermore, HIFs stimulate angiogenesis and support tumor cell survival. Under hypoxic conditions, EGR1 expression in prostate cancer cells increases, and EGR1 directly binds to the HIF1 promoter, causes HIF1 expression, and contributes to tumor angiogenesis (45). EGR1 is reported to directly initiate VEGFA expression in lung cancer cells by binding to the *VEGFA* promoter and to increase angiogenesis by enhancing HIF1α-mediated VEGFA expression (30).

EGR1 is important for the initiation of the growth and migration of vascular endothelial cells and associated angiogenesis because EGR1 acts *via* fibroblast growth factor 2 (FGF2) (46–48), which can launch *EGR1* expression (49). Furthermore, EGR1 may lead to angiogenesis through the NT1–DCC–VEGF pathway (50). Many types of cancer cells secrete extracellular vesicles, which are known to stimulate the migration of vascular endothelial cells by upregulating EGR1. This is an important angiogenesis-enhancing mechanism (51).

The proliferation and migration of lymphatic endothelial cells is another important adaptive response of tumor cells to hypoxia. EGR1 can participate in hypoxia-induced lymphangiogenesis through the VEGF signal transduction pathway (31), but the underlying molecular mechanism needs further investigation.

## Antitumor Effects of EGR1

**Figure 3:**
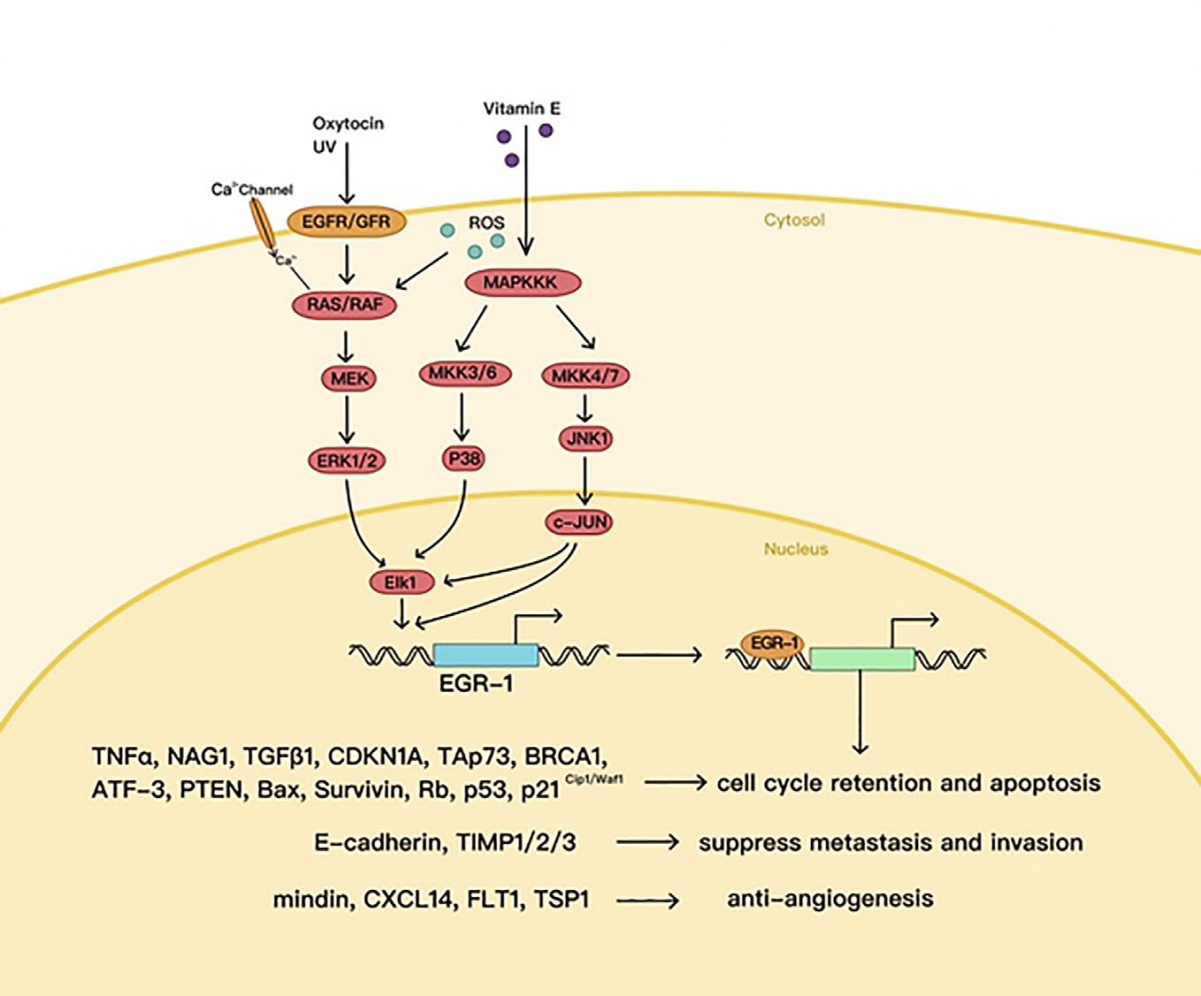
The antitumor effects of EGR1.

In certain cases, EGR1 plays an antitumor part, e.g., in p53 (TP53)-deficient prostate cells, where EGR1 is believed to promote apoptosis by activating TNF-α (52). EGR1 also increases tumor cell apoptosis by directly upregulating tumor suppressors called non-steroidal anti-inflammatory drug (NSAID)-activated gene 1 (NAG1) and PTEN (53–55) (**Figure 3**).

## Mechanisms Whereby EGR1 Induces Tumor Cell Apoptosis

Apoptosis is a process of programmed cell death under physiological conditions, and EGR1 causes tumor cell apoptosis through a variety of mechanisms. One such mechanism is direct binding to promoters of various apoptosis-inducing factors, such as BAX, NAG1, and PTEN, and stimulation of their expression (8, 56, 57). NAG1 belongs to the transforming growth factor β (TGF-β) superfamily and inhibits the growth of tumor cells. Recently, it was found that an EBS is present in the *NAG1* promoter, and EGR1 expression significantly increases NAG1-mediated apoptosis in colon, lung, and liver cancers (53, 54). NSAIDs lead to apoptosis in a COX2-dependent manner by launching the PPARγ–EGR1–NAG1 signaling pathway. In addition, NSAIDs can directly strengthen *NAG1* expression that is mediated by EGR1 and promote apoptosis in a COX2-independent manner (8). In pancreatic cancer cells, δ-tocotrienol triggers EGR1 expression *via* the JNK–c-Jun pathway, and the upregulated EGR1 binds to the *BAX* promoter to initiate the expression of BAX, which causes apoptosis of pancreatic cancer cells (56). JUN is the upstream transcription factor of *EGR1* and is known to directly bind to the promoter region of *EGR1* and to start its expression. In multiple myeloma, EGR1 that is induced by JUN triggers the EGR1–survivin–caspase signaling cascade and drives tumor cell apoptosis (58). *PTEN* is an important tumor suppressor gene, and there is an EBS in the *PTEN* promoter. EGR1 may regulate PTEN expression by targeting the *PTEN* promoter, thus resulting in tumor cell apoptosis (55). Research suggests that vitamin D receptor, EGR1, and p300 synergistically initiate PTEN expression and apoptosis in cancer cells (57).

EGR1 may play a key role in the regulation of DNA repair mechanisms by controlling key effectors, including p53, p21, and BRCA1. As a tumor suppressor, p53 is mainly involved in the monitoring of DNA damage and maintenance of genomic stability and is highly implicated in human cancers. EGR1 can act on the *TP53* promoter and initiate the expression of p53, which in turn further activates EGR1, thus forming a feedback loop (59, 60). *In vitro* studies indicate that EGR1-deficient cells are characterized by p53 inactivation and loss of responsiveness to DNA damage, thereby revealing an important function of EGR1 in upstream transcriptional regulation of p53 (61). In p53-deficient prostate cancer cells, EGR1 is thought to stimulate apoptosis by initiating TNF-α expression (52). P21^Waf1/Cip1^ is the main target of p53 and controls the cell cycle. On the other hand, EGR1 can induce p21^Waf1/Cip1^ expression independently of p53 through ERK and JNK MAPK–ELK1–EGR1 pathways to launch DNA repair and enhance apoptosis, suggesting that EGR1 is a good therapeutic target in p53-mutant tumors (62). As a tumor suppressor, the product of breast cancer susceptibility gene *BRCA1* participates in gene transcription and DNA repair. Three EBSs have been identified in the enhancer region of *BRCA1*, and EGR1 is able to directly bind to the EBSs and to start *BRCA1* gene expression thus initiating DNA repair and inhibiting cancer progression (63).

## Mechanisms Whereby EGR1 Suppresses Tumor Invasion and Metastasis

EGR1 has been reported to inhibit tumor invasion and metastasis in many tumor types. TGF-β1 can downregulate EGR1-induced NSCLC cell EMT, and high expression of EGR1 significantly reduces EMT (64). The mechanism may be related to the regulation of SNAIL, SLUG, and E-cadherin expression by EGR1. In head and neck squamous cell carcinoma, oxytocin is thought to inhibit tumor invasion and metastasis by upregulating EGR1 through an EGFR-and-ERK–dependent pathway (65). The mechanism in question may be related to E-cadherin overexpression. In hepatocellular carcinoma, it has been demonstrated that β-lapachone induces the expression of EGR1, and the latter may suppress invasion and metastasis by affecting the expression of TSP1, SNAIL, and E-cadherin (66). Liu et al. have reported that the use of thalidomide against leukemic cells may inhibit their metastasis, and this phenomenon was attributed to upregulated EGR1 (67). In subsequent studies, it has been revealed that LY294002 inhibits the invasiveness and metastasis of leukemic cells by upregulating EGR1, and this mechanism is independent of the PI3K–AKT pathway (68). Overexpressed EGR1 can significantly repress tumor cell invasion in fibrosarcoma, and the underlying mechanism may be related to increased expression of tissue inhibitor of metalloproteinase 2 (TIMP2), which is regulated by EGR1 (69). Nasopharyngeal carcinoma–associated gene 6 (*NGX6*) is expressed in diverse tumors and is considered a tumor suppressor. EGR1 can directly increase *NGX6* expression by binding to its promoter region and in this way inhibit tumor invasion and metastasis (70). Although EGR1 is known to reduce invasion and metastasis of many types of tumors, the specific mechanism needs to be further researched.

## Mechanisms Underlying the Antiangiogenic Action of EGR1

Angiogenesis is the formation of new blood vessels from the existing vascular system and is necessary for many physiological and pathological processes. Neovascularization provides nutrition and oxygen and removes carbon dioxide and other metabolic waste. When EGR1 is continuously expressed, a variety of antiangiogenic genes are overexpressed, such as *CXCL14*, *TIMP1*, *TIMP3*, and *FLT1*, which inhibit tumor angiogenesis (71). EGR1 can also diminish tumor angiogenesis by upregulating TIMP2 (69). According to colon cancer studies, EGR1 controls mindin expression at the transcriptional level by binding to its promoter. Overexpression of mindin both inhibits the expression of HIF1α and VEGFA in colon cancer cells and reduces VEGFR2 phosphorylation in endothelial cells, resulting in antiangiogenic changes (72). *NGX6* is a metastasis suppressor gene whose functions are related to cell proliferation, cell cycle, and tumor angiogenesis. Recent research suggests that there are overlapping binding sites for Sp-1 and EGR1 in a *NGX6* promoter region and that EGR1 increases *NGX6* expression and decreases tumor angiogenesis (70, 73). Fluorouracil is widely used in many cancer therapies and is believed to upregulate EGR1 through the p38 MAPK pathway; this drug inhibits tumor angiogenesis. Overexpressed EGR1 binds to the thrombospondin 1 (*TSP1*) promoter, enhances TSP1 expression, and diminishes tumor angiogenesis (74, 75). EGR1 can interact with a variety of antiangiogenic factors, such as mindin, NGX6, and TSP1, and may be used as a major target of antivascular therapy to develop relevant drugs.

## Applications of EGR1 in Cancer Treatments

### The Mechanism of Action of the EGR1-and-Radiotherapy Combination

The promoter of *EGR1* contains the characteristic CArG box sequence for SRF binding, which is activated by radiation (76, 77). EGR1 is reported to regulate target genes—after ionizing radiation induces it—e.g., *TNF-α*, *TP53*, *RB*, and *BAX*, and to cause tumor cell growth arrest or cell death (52, 78). Because EGR1 can lead to the activation of apoptosis-associated factors and downregulate survival-related factors to reduce radiation resistance, EGR1 expression in primary tumors is related to the tumor radiation response, and EGR1 expression in irradiated tissues also correlates with residual tumor size and tumor recurrence (79). The CArG box in the promoter of *EGR1* is key to the initiation of the EGR1-related antitumor effect by radiotherapy. Bickenbach K.A. et al. have ligated the CArG box to the transcription start site of *TNF* cDNA—to construct a vector combining the *EGR1* promoter and the tumor-killing gene—and then transfected it into tumor cell lines (2). This vector effectively enhances the killing of radiotherapy-resistant tumor cells by radiation (2). The CArG box in the *EGR1* promoter is an effective anticancer tool for suicide gene therapy combined with radiotherapy and has a great potential to improve the efficiency of antitumor therapies.

### The Mechanism of Action of EGR1 in Combination With Various Antineoplastic Drugs

One of the key mechanisms of action of CD20-targeting drugs is direct stimulation of cell death, but the signal transduction cascade at work here remains unclear. It has been found that CD20-targeting drugs, such as rituximab and obinutuzumab, strengthen EGR1 expression by increasing calcium influx *via* different mechanisms and subsequently lead to cell death. Inhibition of calcium influx by calcium channel blockers (CCBs) can prevent EGR1 induction by CD20-targeting drugs and weaken these drugs’ effects *in vivo* and *in vitro*. By analyzing the actions of CCBs in patients treated with CD20-targeting drugs in the GOYA and REMARC clinical trials, investigators found that patients who received both a CCB and a CD20-targeting drug showed shorter progression-free survival and overall survival (80). These results mean that EGR1 is the key mediator of cell death that is directly caused by CD20-targeting drugs and offer a rationale for EGR1 use as a new predictive quantitative biomarker of therapeutic responses to CD20-targeting drugs (80).

Certain natural compounds affect the regulation of EGR1 expression too. Quercetin initiates the expression of *NAG1* by upregulating EGR1 and mediates apoptosis of colon cancer cells (81). The EGR1-binding site in the promoter region of *NAG1* is the key point in the underlying mechanism. Resveratrol stimulates cancer cell apoptosis by upregulating EGR1 (82). Shi et al. have constructed a suicide-causing gene therapy vector by inserting the *EGR1* promoter upstream of the *GADD45A* cDNA gene, and a combination of this vector and resveratrol inhibited the proliferation of lung cancer cells *in vitro* (83). Studies have shown that resveratrol effectively triggers a suicide-causing gene therapy vector constructed by means of the *EGR1* promoter, and the CArG box in the *EGR1* promoter may be the target site of resveratrol (84). The CArG box in the promoter of *EGR1* can be activated by cisplatin too. Wang et al. have constructed an adenoviral vector that contains the CArG box and human wild-type *TP53* gene; this vector enhances the therapeutic response to the antitumor treatment with cisplatin in NSCLC cell-transplanted mice (85). The suicide-causing gene therapy vector constructed by means of the *EGR1* promoter sequence is known to be activated by many compounds, such as resveratrol and cisplatin; this knowledge may point to a new strategy for targeted cancer treatment. The investigation into the mechanism of action and molecular interactions of EGR1 in tumors should lead to new breakthroughs in antitumor therapies.

### Prognostic Value of EGR1

In lung cancer, the downregulation of PTEN and EGR1 expression is related to tumor drug resistance (86, 87). The expression of EGR1 predetermines PTEN levels, which may predict treatment resistance resulting from PTEN pathway loss. Accordingly, low EGR1 levels are associated with poor prognosis (86). Besides, the EGR1 expression level is closely related to the pathological features and prognosis of patients with nasopharyngeal carcinoma, and high EGR1 expression correlates with a low histopathological grade and good clinical prognosis (88). EGR1 expression is higher in pituitary adenomas than in healthy control tissue samples, and EGR1 levels in invasive pituitary adenomas are even higher (89). EGR1 can serve as a prognostic factor in pituitary adenomas (89). In gastric cancer, EGR1 status is associated with malignancy, tumor stage, and prognosis (13, 14). EGR1 is of great value in the prognosis of the above tumor types, but the specific mechanism of action here and the prognostic value of EGR1 in other cancers need to be further explored.

## Discussion

In summary, EGR1 is a key molecule implicated in many signaling pathways. In some cases, EGR1 is a tumor suppressor that helps to monitor DNA damage, promotes tumor cell apoptosis, and enhances the anticancer effects of radiotherapy and chemotherapy. By contrast, in certain tumor microenvironments, such as hypoxic ones, EGR1 expression increases to maintain tumor cell survival, proliferation, and metastasis and tumor angiogenesis. Abnormal EGR1 expression is common among diverse human tumors, but its role requires further research. At present, the specific mechanism underlying the EGR1 “duality” is still unclear but may be linked with the following: Sp-1 expression (70), the expression of other EGR family members (90), the EGR1 self-inhibition zone, and other factors (91). Several of the EGR1 downstream target genes have multiple overlapping SP1 and EGR1 binding sites in their proximal promoter regions. The regulatory relationship in this overlapping area is complex, in some genes the two factors are synergistic, and antagonistic in others (70, 92, 93). The duality of EGR1 is related to the regulatory relationship between the two factors. EGR transcription factor family includes EGR1, EGR2, EGR3, and EGR4. All of them have similar zinc finger structures and can bind to the same EBS. Wang et al. have reported that the EGR family genes can be verified after EGR4 knockdown in pGCs (94). Tourtellotte et al. showed that EGR4 loss upregulated EGR1 and slightly altered EGR2 and EGR3 (95). When any one of the coding genes in the EGR family is knockdown, there will be a competitive effect on the expression of the other three. NAB1 and NAB2 are repressor proteins of EGR1, which can bind to the zinc finger structures of EGR1. EGR1 can bind to the promoter region of NAB1 and NAB2 to induce their expression. When EGR1 was overexpressed, EGR1 upregulated the expression of NAB1 and NAB2, and negative feedback regulated its own expression. Thus the expression of downstream genes is regulated by EGR1 itself (91, 96). Thus, the mechanisms of action of EGR1 in various cancers require additional exploration. Determining and exploiting the involvement of EGR1 in cancer will help develop new therapies for patients with malignant tumors.

## Author Contributions

BW carried out the primary literature search, drafted and revised the manuscript, and participated in discussions. HG, HY, HX, and GZ helped modify the manuscript. All authors contributed to the article and approved the submitted version.

## Funding

This work was supported by National Nature and Science Foundation of China (81772684 and 81672505), Scientific Research Foundation of Jilin province (20200201469JC, 20200404101YY, 20200201613JC, 20200201388JC, 20190701042GH, 20180101152JC), Health and Wellness Technology Enhancement Project of Jilin Province (2019J004 and 2017J045), Research and Planning Project of the 13th Five-Year Science and Technology Project of Jilin Provincial Department of Education (JJKH20201077KJ and JJKH20180191KJ) and Interdisciplinary Innovation Project of First Hospital of Jilin University (JDYYJC001).

## Conflict of Interest

The authors declare that the research was conducted in the absence of any commercial or financial relationships that could be construed as a potential conflict of interest.
